# Autoantibody:Autoantigen Competitor Decoys: Application to Cardiac Phenotypes

**DOI:** 10.3389/fimmu.2022.812649

**Published:** 2022-01-28

**Authors:** Timothy Cardozo, Lila Cardozo, Mohamed Boutjdir

**Affiliations:** ^1^ Department of Biochemistry and Molecular Pharmacology, New York University (NYU) Grossman School of Medicine, New York, NY, United States; ^2^ Department of Medicine, New York University (NYU) Grossman School of Medicine, New York, NY, United States; ^3^ Department of Medicine, Cell Biology and Pharmacology, State University of New York Downstate Medical Center, New York, NY, United States; ^4^ Cardiovascular Research Program, VA New York Harbor Healthcare System, New York, NY, United States

**Keywords:** autoantigen, autoantibody, therapeutics, decoy, autoimmune, long QT

## Abstract

Autoimmune diseases are often associated with autoantibodies that abnormally target self-antigens (autoantigens). An intuitive therapeutic strategy for diseases caused by aAbs is to design decoys, or soluble molecules that target the antigen combining site of these aAbs, thereby blocking binding of aAb to self-antigen and subsequent tissue damage. Here, we review the known decoy molecules of these types, discuss newer technological opportunities afforded by monoclonal antibody and structural biology advances, and discuss the challenges to this approach. Recent opportunities relevant to this approach for cardiac phenotypes, specifically Ro-associated long QT syndrome, are discussed.

## Common Autoantigens

Autoantibodies (aAbs) have historically been of interest primarily as diagnostic markers of autoimmune diseases, most notably rheumatologic connective tissue diseases (systemic lupus erythematosus, dermatomyositis, Sjogren’s Syndrome, etc.). As molecular techniques and disease diagnostic criteria have improved, a growing number of autoantigens have been recognized, which can be browsed and analyzed in the aAgAtlas database (1). This database reveals that intracellular nucleic acid binding proteins and signaling molecules are the most likely to behave as autoantigens. Indeed, the historical flagship Ro (SSA) and La (SSB) autoantigens are proteins in these classes. Ro, in particular, was initially identified serologically, but was later found to have two independent components, both of which are autoantigens: SSA/Ro60, a ribonucleoprotein, and Ro52, also called TRIM21, an E3 ubiquitin ligase enzyme. Seropositivity to both these autoantigens is highly prevalent in the general population and often specific for symptomatic connective tissue disease, whereas the sensitivity and specificity of the remaining large list of autoantigens ranges widely from nearly 100% specific for certain diseases (anti-double-stranded DNA, Smith (Sm) antigen) to nearly 100% sensitive for disease (anti-nuclear antigen, ANA). Remarkably, despite this immense body of knowledge on Ro (SSA) and La (SSB) aAbs in human disease, very few molecular mechanisms connecting aAbs to their epitopes *and* to their disease phenotypes have been unveiled. Indeed, epitope mapping of sera from SSA/Ro60-positive patients with connective tissue disease on the 3D structure of SSA/Ro60 did not reveal a single, convincing immunodominant B-cell epitope, and diagnostic value for any single SSA/Ro60 B-cell epitope (auto-epitope) has not been established (2). This scenario remains true for all the Ro (SSA) and La (SSB) autoantigens. The absence of a molecular connection between aAb and target auto-epitope and subsequent connection between the aAb-autoepitope interaction and disease phenotype clearly has contributed to obfuscating the most direct and intuitive translation of this knowledge into therapy: the decoy approach. Here, we review in further detail the components of the decoy approach and current progress in this direction.

## Overview of the Decoy Approach

**Figure 1:**
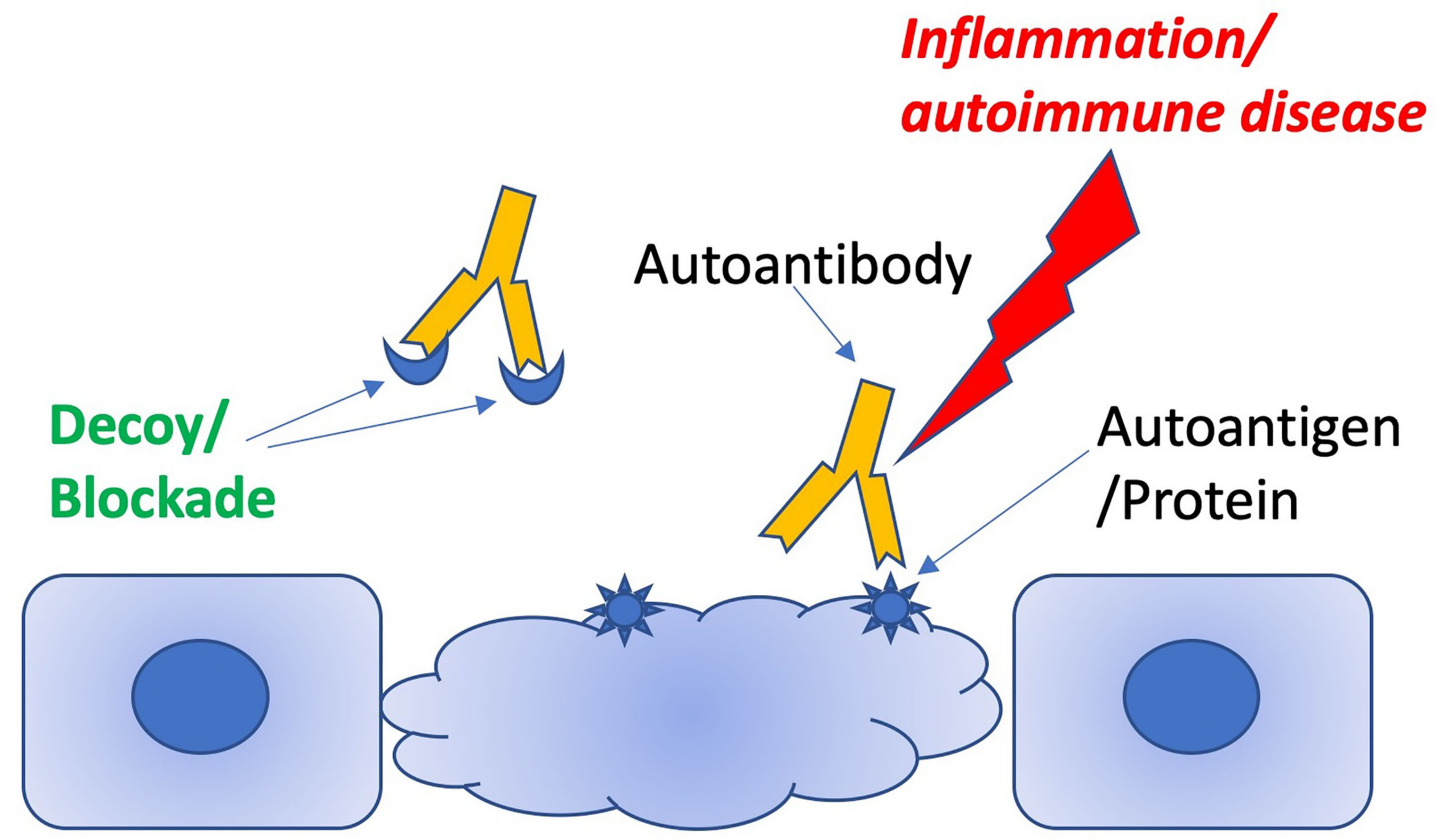
Schematic illustrating how decoy peptides can distract pathogenic antibodies from targeting autoantigens.

The aAb decoy therapeutic approach is conceptually straightforward. aAbs are hypothesized to cause a disease phenotype by binding to autoantigens and triggering inflammation. Autoantigens may be soluble and circulating, leading to inflammation *via* the formation of autoantibody-autoantigen immune complexes (3), or they may be displayed on cell surfaces, potentially targeting autoantibody-mediated inflammation to specific cells and tissues (**Figure 1**). A soluble molecule that mimics the autoantigen and successfully competes with the native autoantigen for the aAb should prevent this engagement and therefore prevent the inflammation in either scenario. A by-product of demonstrating reversal of the phenotype is that the aAb is proven to cause the disease/phenotype. There are two sub-mechanisms within this scenario: 1) the autoantigen may be obvious (e.g. double-stranded DNA, dsDNA), or 2) the autoantigen may be a cryptic self-molecule and the result of molecular mimicry, namely serendipitous B-cell epitope cross-reactivity between the native autoantigen and the pathogenic auto-epitope (e.g. anti-Ro antibodies and ion channel epitopes).

## Koch’s Postulates, as Applied to aAbs

The decoy molecule approach to treating aAb-elicited disease necessarily depends on whether aAbs actually cause disease. Conversely, one would expect a decoy molecule to mitigate only the phenotype caused by the aAb and not the broader disease. This is an especially complex issue for autoimmune diseases, as they are almost always a collection of diverse phenotypes and biomarkers, no single one of which is highly predictive of the presence of the disease. A useful conceptual framework to evaluate whether aAbs cause a particular disease phenotype is to apply Koch’s postulates for infectious diseases to the question: namely 1) the aAb must be present with the clinical phenotype; 2) it must be detectable in the blood or tissue; and 3) it must replicate the disease in an experimental model, either an animal or a tissue culture system. Due to the nature of polyclonality of B-cell responses in mammals, a critical further requirement is that the human aAb, which is present with the clinical phenotype and detectable in blood of patients with autoimmunity, must either itself cause the phenotype/disease in an animal or cell or target the same B-cell epitope as any presumably orthologous animal antibody that causes the phenotype/disease in the animal. Notably, this requirement should be more readily met at present than in the past due to advances in molecular structure determination (X-ray crystallography, cryo-EM). In addition, for certain autoantigens such as double-stranded DNA, epitope diversity may be less than for protein autoantigens, and, as such, 3D confirmation may not be necessary. Indeed, Koch’s postulates are arguably met for lupus glomerulonephritis caused by anti-double-stranded-DNA aAbs (anti-DS): anti-DS are present in the blood of patients with systemic lupus erythematosus and lupus nephritis [100% specific, variably sensitive (4)] and a mouse antibody (R4A) causes glomerulonephritis in a mouse model (5), as well as several other autoimmune phenotypes/aAbs (4). However, from a strict 3D structure point of view, the requirement has not been met: e.g. the 3D structure of the complex of a human anti-DS mAb from Lupus nephritis patients has not been proven to bind the same 3D epitope as R4A. Decoy therapeutics, e.g. a molecule that blocks the binding of R4A or human anti-DS to DNA can satisfy Koch’s postulates: if they prevent of ameliorate nephritis, Koch’s postulates are met. This test has been met in the animal (5), but has not yet been clinically proven. Thus, decoy therapeutics can address a major knowledge gap in the field simply by their testing in human subjects afflicted by the phenotype and bearing the aAbs. If the phenotype is ameliorated or suppressed by the decoy, the hypothesis that the phenotype is caused by the aAbs is proven.

## 3D Structures of Auto-Antigen/aAb Complexes

**Figure 2:**
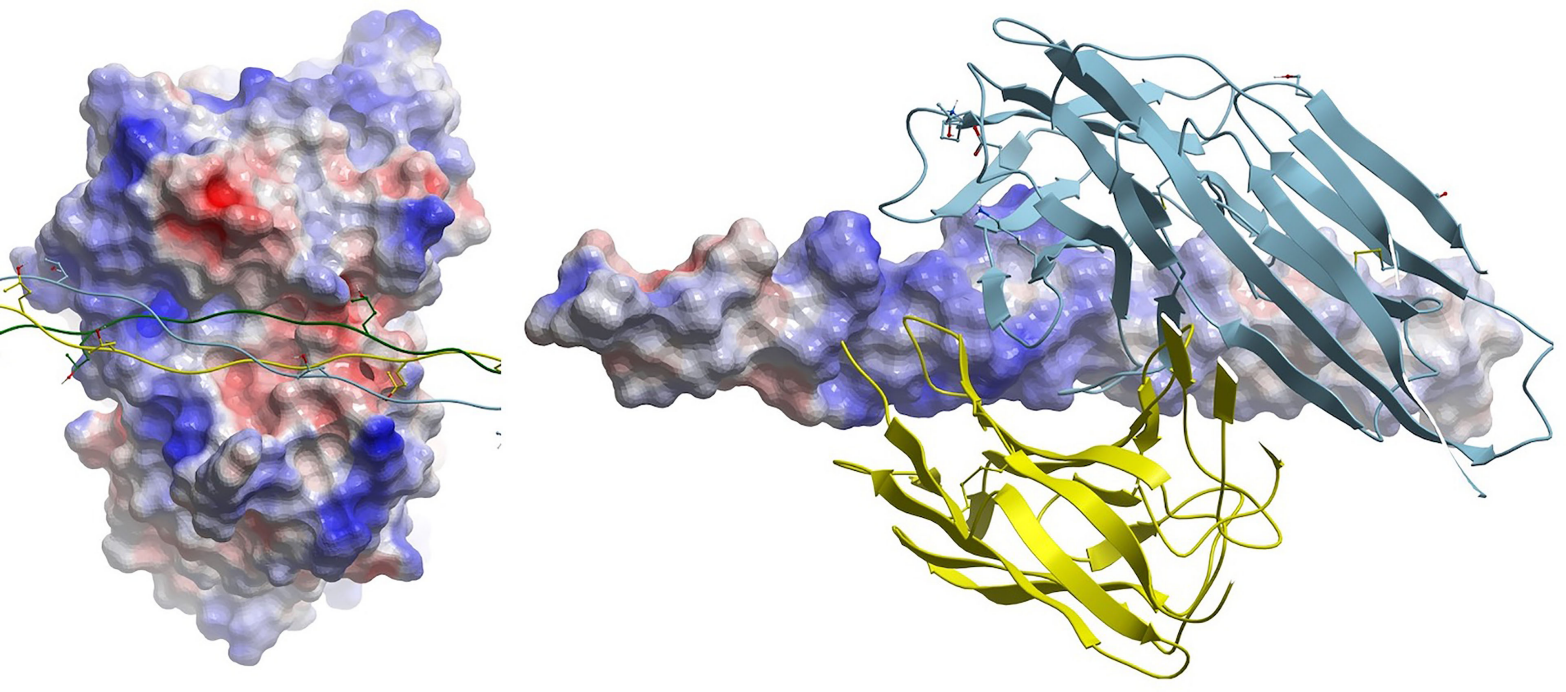
(Left) Electrostatic surface of antigen (epitope) combining site of mAb CIIC1. Triple-helical collagen C1 epitope is shown as a ribbon with some side chains displayed in stick form. (Right) Cognate electrostatic surface of epitope. The mAb is shown in ribbon style.

Koch’s postulates emphasize the importance of 3D auto-epitope structure in the causality argument for aAbs in autoimmune disease. However, remarkably few auto-antigen/aAb complexes are available, and none are for cross-reactive epitopes, only native auto-epitopes (**Table 1**). Collagen-induced arthritis (CIA) represents the best data package to meet Koch’s postulates that also contains a crystal structure of a monoclonal autoantibody bound to its target B-cell epitope. CIA mAbs elicit arthritis in genetically engineered mice (12), and the structure of an mAb bound to the C1 epitope of type II collagen has been resolved (9). However, reversal of mAb-elicited arthritis by the exact peptide epitope seen in the crystal structure, infused or injected as a decoy, has not been reported. The electrostatic surfaces of the interface between the C1 epitope and the mAb do not appear to be unusual, with common electrostatic and hydrophobic contact areas (**Figure 2**), so theoretically, the decoy approach should be demonstrable for at least one observable phenotype within CIA, with the major challenges being dosing and timing: decoys may be ineffective or effective alternatively during development vs. maintenance of the phenotype, wherein the latter may or may not have broadened the immunopathogenesis to establish independent pathogenic T-cell responses. Indeed, soluble MHC-peptide complexes have been demonstrated to reduce symptoms in the acute phase of the CIA model (13).

**Table 1:** Experimental 3D structures of autoantibody : Autoantigen complexes.

Autoantigen/Disease	aAb	PDB code	Reference
Thyrotropin Receptor/Autoimmune Hypothyroiditis	K1-70	2XWT	(6)
Fc/Rheumatoid Arthritis	Rheumatoid Factor	1ADQ/5XMH	(7, 8)
Type II collagen/Rheumatoid Arthritis	M2139, CIICI	4BKL/2Y5T	(9, 10)
Acetylcholine Receptor/Myasthenia Gravis	Mab 198	2JRV	(11)

Indeed, 3D structures of the Ro (SSA) and La (SSB) have been elusive, although reliable 3D models of human SSA/Ro60 have been obtained (14), and Ro52 might now have been reliably visualized by artificial intelligence breakthroughs in protein structure prediction from sequence alone (15, 16). Mapping of linear B-cell epitopes recognized by human patient sera onto the SSA/Ro60 structure revealed some patterns, but no clear immunodominant epitope and no evidence meeting Koch’s postulates (2). This absence of 3D structure-activity relationships further emphasizes the need for clinical tests of aAb decoy molecules that mimic the auto-epitope to both generate new therapeutics for autoimmune diseases and prove that a specific aAb causes the disease or phenotype.

## Challenges of Mimicking Native Autoepitopes

To date, molecules mimicking the linear B-cell autoepitopes themselves have been extensively pursued for diagnostic purposes, but not been sufficiently informative to be of diagnostic value (2), unless one considers dsDNA to be a linear B-cell epitope. Diagnostic probe molecules are most easily pursued as short peptides, i.e. *via* peptide array surveillance, so some of the failure may be due to inability to capture conformational or heterogeneous (e.g. ribonucleoprotein or glyco-peptide) epitopes. However, the failure to isolate peptides or similar molecules that sensitively or specifically correlate with specific rheumatologic phenotypes in patients may also reflect the complexity of autoimmune pathogenesis, wherein defects in cellular processes may be the primary insult, immune cellular responses may be the main mediators, and aAbs are more surrogate than cause (17). Indeed, serologic anti-Ro aAbs may not be a traditional memory B-cell response, and instead be constantly renewing short-term clonotypes (18), making visualization of the target aAb, either structurally or by monoclonal antibodies, elusive. Furthermore, even conformational epitopes may be further complicated by their presentation of different conformations in the native intracellular context as compared to exposed on an assay solid support (19). Nevertheless, significant effort has been made previously to develop therapeutic decoy molecules that compete with native auto-antigens, because some narrowly-defined, autoimmune phenotypes and corresponding narrowly specific aAbs (e.g. anti-DS) have strong arguments for being causally related (5, 20). The earliest report of such a molecule harnessed high-throughput RNA screening to mimic an insulin receptor auto-epitope responsible for extreme insulin resistance Type B (21). The most convincing *in vivo* demonstration of efficacy was for decoy molecules to block anti-DS elicitation of Lupus nephritis, which was demonstrated with multiple molecules, including a repurposed HIV protease inhibitor drug (5, 20). These results suggest that at least some, if not all, aAbs can cause disease phenotypes and therefore be targeted *via* the decoy approach, and that amenable aAb-phenotype pairs were likely narrowly specific, both in aAb specificity and in phenotype definition. As expected, this narrow specificity defines most reports of decoy molecules to date. Most have been peptides (9 reported) or RNA (4), although more complex molecules such as exosomes and platelets have been reported (**Table 2**). None of the flagship autoantigens, such as Ro/SSA, La/SSB or Sm, are present, suggesting that the breadth of their aAb specificity and the diversity of their phenotypes represents a challenge. Clearly, a major challenge of the decoy approach for some auto-epitopes is molecular engineering of a molecule that can mimic conformational or heterogenous epitopes, but, nevertheless, some decoys may succeed as linear short peptides alone for a suitable aAb-phenotype pair. Engineering an affinity of the decoy molecule to be higher than the affinity of the autoantibody for its autoantigen may be a related challenge: for example, engineered peptides have not historically competed well with the affinity of monoclonal antibodies in tissue-targeted drug delivery efforts (30). Nevertheless, although 3D structure is not part of the story, the decoy approach has been arguably demonstrated for antiphospholipid syndrome (aPL), wherein the N-terminal domain of ß2-glycoprotein-1 infused into a mouse model of anti-phospholipid syndrome thrombosis, which was elicited with purified IgG from aPL patients, reduced the thrombotic phenotype (25).

**Table 2:** Reported Synthetic Autoantibody Decoys/Autoantigen Mimics.

Autoantigen/Disease	aAb	Decoy molecule	Reference
Insulin Receptor/extreme insulin resistance Type B	MA20	RNA	(21)
DS DNA/Lupus nephritis	R4A	Peptide/HIV protease inhibitor compound	(5, 20)
Acetylcholine Receptor/Myasthenia Gravis	Mab 198	RNA/Peptide	(22–24)
ß2-glycoprotein-1/Antiphospholipid syndrome	aPL	Peptide/platelets	(25)
Myelin associated glycoprotein (MAG)/Multiple Sclerosis	Anti-MAG IgM	Peptide/glycopolymer	(26)
Collagen type 17/Bullous Pemphigoid	Anti-COL17	Peptide	(27)
Angiotensin 1 receptor/Hypertension	Anti-AT1R	Peptide	(26)
Thyrotropin Receptor/Hypothyrodism	Anti-TSHR	Exosomes	(28)
Platelet Membrane/Autoimmune Thrombocytopenia	Anti-Platelet	Platelet Membrane-coated nanoparticles	(29)

## Cross-Reactive Auto-Epitopes

Cross-reactivity or molecular mimicry represents another angle for the decoy approach. For example, it has been suggested that a specific candidate auto-epitope in SSA/Ro60 becomes pathogenic *via* its mimicry of an Epstein Barr Virus (EBV) epitope (2). This presents additional challenges and considerations for the decoy approach and, not surprisingly, more limited efforts have been dedicated to develop therapeutic decoy molecules for cross-reactive epitopes. For example, most aAbs in autoimmune diseases are not pathogenic aAbs: e.g. a significant fraction of the general population exhibit anti-Ro antibodies yet exhibit no symptoms. It is unknown whether all aAbs are pathogenic, but require exposure of intracellular autoantigens to elicit pathology, or whether a small fraction target select epitopes on the autoantigen that are cross-reactive with cell surface or extracellular gene products and produce deleterious effects by engaging the signaling pathways associated with these cross-reactive targets. It has been hypothesized for SSA/Ro60 that the initial aAb is the EBV mimic, with the diversity of anti-SSA/Ro60 aAbs that follow and are observed variably across patients being due to epitope spreading (2). Notably, this epitope spreading hypothesis suggests that additional cross reactivities are then generated. Most notable of the latter examples are the reports that anti-SSA/Ro60 antibodies cross react with the L-type calcium channel as a potential cause of neonatal Lupus (also known as congenital heart block) (31–33) and reports that certain anti-Ro52 antibodies cross-react with the hERG channel to elicit long QT syndrome (LQTS) (34–36). The cross-reactive hypothesis thus suggests that an elusive, narrow, possibly clonal, fraction of anti-Ro antibodies are susceptible to decoy therapeutics, with respect to specific cardiac phenotypes. Two methods to target this species are 1) to isolate the monoclonal antibodies that cross react with the pathogenic targets (e.g. cross react with hERG epitopes, for example) and 2) designing decoy molecules based directly on knowledge of the epitopes of the cross-reacting pathogenic targets, which should then target only the cross-reactive auto-antibodies, leaving all other anti-Ro antibodies unperturbed. In the latter case, if the cardiac phenotype is improved, Koch’s postulates may be achieved for that specific aAb and cardiac phenotype pair.

## Autoimmune LQTS as a Decoy Therapy Opportunity

Given the experience with anti-DS aAbs causing the specific phenotype of Lupus nephritis and anti-C1 mAbs causing CIA, the most specific cardiac phenotype, which can be recapitulated in an animal model and non-invasively detected (e.g. long QT on the electrocardiogram) may be a good target to advance a decoy approach. Several findings to date support the autoepitope-phenotype pair of cross-reactive anti-Ro52/hERG-K^+^ aAbs and LQTS as an attractive target for a decoy therapeutic approach. LQTS is an electrocardiac disorder characterized by abnormal prolongation of the heart rate-corrected, QT interval ([QTc], traditionally >440 ms; currently, >470 ms for men, and >480 ms for women) on the electrocardiogram (ECG) (37). LQTS predisposes to life-threatening ventricular arrhythmias (VAs), specifically Torsades de Pointes (TdP) (37–39), which is a polymorphic ventricular tachycardia that can rapidly degenerate into ventricular fibrillation (VF) and cause sudden cardiac death (SCD) (1). Autoimmune-associated LQTS has recently been recognized as a clinical phenomenon (40, 41), which increases the health impact of an eventual decoy therapeutic for this phenotype. In addition, there are several preclinical advantages to the development of a decoy for autoimmune-associated LQTS. First, LQTS can be reliably elicited in the guinea pig *via* Ro52 immunization (36), which is an ideal animal model, correlated with *in vitro* and *ex vivo* orthogonal models (including electrophysiologic readouts) (36, 42–44), for testing the decoy hypothesis. Second, the target autoepitope and cross-reactive host antigen, the human *ether-à-go-go* related gene K^+^ channel (hERG-K^+^) for anti-Ro52 aAbs is strongly suspectedv. Finally, should a suitable decoy molecule be developed, it may be non-invasively tested *in vivo* in this model *via* the ECG, and minimally invasively *via* serum Ab profiling. Such a decoy may prove Koch’s postulates for another aAb-autoimmune phenotype and serve as a prototype for decoy therapeutics generally to build on the anti-DS model and other prior attempts (**Table 2**) with a purely peptide model and the first decoy therapeutic for the flagship Ro (SSA) and La (SSB) class of autoantigens.

## Projected Safety and Patho-Biological Issues With the Decoy Approach

Although the aAb-targeted, autoantigen-competitive decoy approach to novel therapies and proof of Koch’s postulates in autoimmune diseases is a theoretically very attractive concept and has a strong publication track record from a preclinical point of view (see **Table 2**), the complete absence in the literature of clinical data on this approach raises some concerns. While this could be due to the “valley of death” of therapy development, namely the high financial cost of preclinical optimization and clinical proof of concept, there are several obvious biological concerns that can be inferred (45). First, a decoy molecule may be immunogenic and elicit *more* of its cognate aAb. This concern seems minimal since the nature of the disease is a maximally stimulated B-cell clone or clones anyway, so additional autoantigen is unlikely to stimulate the system further. Second, a decoy molecule with multiple valencies risks immune complex formation, with attendant inflammatory tissue pathology. This is easily avoided by restricting decoy molecules to one auto-epitope per particle. Finally, pathogenic aAbs may be too numerous to sequester without very high doses of decoy molecules. This may be a pharmacokinetic challenge, but was not evident in prior preclinical results (**Table 2**). Overall, there does not seem to be a compelling case against the decoy approach to therapies for narrowly defined aAb-autoimmune phenotype pairs.

## Conclusions

The concept of designing molecules to block aAbs at their auto-epitope binding sides in their variable domains is highly attractive for multiple reasons, namely novel drugs for autoimmune diseases, proof of Koch’s postulates for autoimmune phenotypes and stratification of patients by molecular factors. That no clinical candidate exploiting this mechanism has emerged for nearly 30 years of reports in the literature for this approach is puzzling. Advances in monoclonal antibody technology, *in vitro* evolution molecular design (e.g. phage display) and structural biology techniques offer major advantages to determine if this therapeutic strategy is valid for autoimmune diseases that prominently feature autoantibodies. Clinical trials of suitably engineered decoy molecules may be the only way to validate and realize the potential of this approach.

## Author Contributions

TC and MB conceived the study. LC and TC collected the data. TC wrote the manuscript. TC, MB and LC edited the manuscript. All authors contributed to the article and approved the submitted version.

## Funding

This work was supported by grant funds from the Biomedical Laboratory Research and Development Service of Veterans Affairs Office of Research and Development (Merit Review Grant I01 BX002137) to MB and the U.S. Department of Veterans Affairs Technology Transfer Program to MB and TC.

## Conflict of Interest

The authors declare that the research was conducted in the absence of any commercial or financial relationships that could be construed as a potential conflict of interest.

## Publisher’s Note

All claims expressed in this article are solely those of the authors and do not necessarily represent those of their affiliated organizations, or those of the publisher, the editors and the reviewers. Any product that may be evaluated in this article, or claim that may be made by its manufacturer, is not guaranteed or endorsed by the publisher.
